# Representation of #CAMHS on social media platform TikTok

**DOI:** 10.1192/bjo.2021.645

**Published:** 2021-06-18

**Authors:** Preetisha Chadee, Sacha Evans

**Affiliations:** Great Ormond Street Hospital

## Abstract

**Aims:**

The video-based free social media app, TikTok, has grown in popularity during the COVID-19 pandemic, with half of British children using Tik Tok regularly. With more than 2 billion downloads, it was the most downloaded app of 2020. Child and Adolescent Mental Health Services (CAMHS) is currently found on TikTok via the hashtag #CAMHS. The aim of this study was to explore how CAMHS is represented on TikTok through reviewing the hashtags associated with CAMHS and exploring the themes of videos with the #CAMHS hashtag.

**Method:**

The Tik Tok app was downloaded and a search for the hashtags which featured the word #CAMHS was undertaken. A thematic analysis of the top 100 most popular uploaded videos featuring the #CAMHS was conducted. The number of likes, views and shares of the videos featuring each theme was recorded.

**Result:**

Videos with the hashtag #CAMHS had 203.9 million views, followed by: #camhsmeme(s) totalling 43.1 million views, #camhsjokes with 21.4 million views and #camhskids, 12.5 million views. The top 100 most popular videos represented 24% of total viewed videos with the hashtag #CAMHS.

The most popular recurrent themes associated with the hashtag #CAMHS in our sample were: raising awareness of mental health symptoms and management (40% of videos), reference to self-harm (27% of videos) and negative perception of CAMHS (27% of videos).

Raising awareness of mental health symptoms and management had the most likes (3,694,700) and views (17,435,900). This was followed by videos with themes of reference to self-harm (3,006,300 likes and 14,382,700 views). The most shared themes were: reference to suicide (shared 56,763 times) and videos which portrayed a theme of negative perception of CAMHS (40,628 shares). Videos with themes of a negative perception of CAMHS also garnered 1,762,500 likes and 8,666,900 views.

**Conclusion:**

CAMHS is actively represented on TikTok through freely accessible unregulated videos. Videos with themes of raising awareness of mental health symptoms and management can potentially allow young people to share their experiences. Nonetheless, popular hashtags such as #CAMHSmemes and #CAMHSjokes, as well as videos featuring themes of negative perception of CAMHS, could potentially undermine the reputation of CAMHS to existing and future service users. The content of these videos should be taken seriously by CAMHS clinicians as it can potentially provide an insight into service users’ experiences of CAMHS on a scale that has not been observed before. Presently these videos are not screened or modulated by the NHS CAMHS service.

